# Association between decreased taurine levels in the anterior cingulate cortex and restricted and repetitive behaviors in autism spectrum disorder: a cross-sectional study

**DOI:** 10.3389/fpsyt.2025.1700059

**Published:** 2025-12-11

**Authors:** Akihiro Minami, Kiwamu Matsuoka, Masato Takahashi, Kazuya Ueda, Hiroki Ohnishi, Yuka Fujimoto, Hiroaki Yoshikawa, Rio Ishida, Yuhei Takado, Jamie Near, Yuya Yamatani, Toshiteru Miyasaka, Yumi Tai, Tomoko Ochi, Toshihiro Tanaka, Takashi Okada, Nakao Iwata, Manabu Makinodan

**Affiliations:** 1 Department of Psychiatry, Nara Medical University, Kashihara, Japan; 2 Department of Psychiatry, Fujita Health University School of Medicine, Toyoake, Japan; 3 Institute for Quantum Life Science, National Institutes for Quantum Science and Technology, Chiba, Japan; 4 Physical Sciences, Sunnybrook Research Institute, Toronto, ON, Canada; 5 Department of Medical Biophysics, University of Toronto, Toronto, ON, Canada; 6 Division of Central Radiology, Nara Medical University Hospital, Kashihara, Japan; 7 Department of Diagnostic and Interventional Radiology, Nara Medical University, Kashihara, Japan; 8 Division of Transformative Psychiatry and Synergistic Research, International Center for Brain Science, Fujita Health University, Toyoake, Japan; 9 Department of Neuropsychiatry, Faculty of Life Sciences, Kumamoto University, Kumamoto, Japan

**Keywords:** autism spectrum disorder, restricted and repetitive behaviors, anterior cingulate cortex, taurine, glutathione

## Abstract

**Introduction:**

Children with autism spectrum disorder (ASD) often experience reduced quality of life due to core autistic traits, such as restricted and repetitive behaviors (RRBs), yet no pharmacological treatments have been established to date. Oxidative stress, a potential contributor to ASD pathology, may reduce taurine and glutathione (GSH) levels. Although animal studies have reported altered antioxidant levels, studies investigating the brain antioxidant levels in individuals with ASD remain limited. This study investigated whether reduced antioxidant levels in the anterior cingulate cortex (ACC), a region consistently characterized by functional and metabolic abnormalities in individuals with ASD, and closely associated with RRBs.

**Methods:**

A total of 44 children with ASD and 40 typically developing controls were enrolled in this study. Diagnoses were confirmed using the Autism Diagnostic Observation Schedule-Second Edition (ADOS-2). Magnetic resonance spectroscopy was used to quantify taurine and GSH levels in the ACC. Statistical analyses were conducted to compare metabolite levels between the groups and assess associations with ADOS-2 subscale scores.

**Results:**

The ASD group exhibited significantly lower taurine levels, whereas GSH levels remained unchanged. Taurine levels were negatively correlated with RRBs but not with social affect.

**Discussion:**

These findings suggest that reduced taurine levels in the ACC of children with ASD, alongside unchanged GSH levels, may indicate distinct biosynthetic pathways and functional roles of these metabolites in oxidative stress defense mechanisms associated with ASD pathology. Taurine depletion may disrupt physiological processes associated with RRBs and could serve as a potential therapeutic target for symptom management.

## 1 Introduction

Autism spectrum disorder (ASD) is a neurodevelopmental condition characterized by impairments in social interaction and communication, along with restricted and repetitive behavior (RRB) (1). According to a survey conducted by the Centers for Disease Control and Prevention, ASD affects approximately 1 in 54 individuals (2). These core features are associated with a reduced quality of life in affected individuals (3, 4). Ongoing neuroimaging research aims to elucidate the underlying pathological mechanisms of ASD (5, 6). Several studies have indicated that individuals with ASD may exhibit both functional (7, 8) and metabolic abnormalities in the anterior cingulate cortex (ACC) (9–11), which is a region strongly associated with RRBs (12, 13). Investigating the molecular targets associated with ASD pathology in the ACC may facilitate the identification of potential pharmacological candidates for symptom management.

Growing evidence indicates that oxidative stress contributes to the pathogenesis of ASD (14–17). The maternal immune activation model, a widely used animal model manifesting ASD-related behavioral abnormalities, has demonstrated that prenatal exposure to immune challenges induces oxidative stress (18). Oxidative stress occurs when the production of reactive oxygen species exceeds the antioxidant defense capacity, resulting in cellular damage (19). BTBRT + Itpr3tf/J (BTBR) mice, which exhibit ASD-like behavioral phenotypes, have reduced serum glutathione (GSH) levels (20). A meta-analysis further demonstrated that individuals with ASD have decreased blood antioxidant levels (21). However, only a few studies have examined antioxidant levels in the living brains of individuals with ASD using magnetic resonance spectroscopy (MRS) (22). These studies focused exclusively on GSH and reported no significant changes in brain levels (23–26).

Taurine (Tau) is a β-amino acid with well-established cytoprotective properties, including functions in energy metabolism, inhibitory neurotransmission, calcium homeostasis, and antioxidative defense (27). Notably, Tau modulates antioxidant defense networks both directly and indirectly (28). It scavenges free radicals (29, 30), reduces lipid peroxidation (31–33), and enhances superoxide dismutase activity (34, 35). Tau is primarily acquired through dietary sources, such as beef, chicken, fish, and shellfish, but can also be synthesized endogenously from sulfate in mammals (36–38). Selective eating behaviors in individuals with ASD may contribute to low Tau levels (39). This hypothesis is supported by studies reporting decreased blood Tau levels in children with ASD compared to typically developing (TD) controls (40–42); however, some studies have found no significant differences (43).

To our knowledge, no studies have measured Tau levels in the brains of individuals with ASD using MRS, with reports to date limited to findings in a model of maternal autoantibody exposure in rats (44). Examining brain levels of Tau and GSH may provide insights into the mechanisms of oxidative stress underlying ASD. The present study is the first *in vivo* investigation to test the hypothesis that children with ASD experience oxidative stress, as indicated by decreased brain levels of Tau and GSH, which may be associated with core autistic traits.

## 2 Materials and methods

### 2.1 Participants

A total of 44 children with ASD were recruited from the outpatient clinic of the Department of Psychiatry at Nara Medical University Hospital and an affiliated psychiatric clinic. Diagnoses were established by two trained psychiatrists according to the criteria of the Diagnostic and Statistical Manual of Mental Disorders, Fifth Edition, and the Japanese version of the Autism Diagnostic Observation Schedule-Second Edition (ADOS-2) (45). Among the children with ASD, nine were diagnosed with comorbid neuropsychiatric conditions, including attention deficit hyperactivity disorder (ADHD) (n = 6), adjustment disorder with tic disorder (n = 1), obsessive–compulsive disorder (n = 1), and learning deficits (n = 1). Psychotropic medications were prescribed to 18 children with ASD during the study period, including antidepressants (n = 4), hypnotic agents (n = 9), antipsychotics (n = 11), anxiolytics (n = 3), ADHD medications (n = 5), and mood stabilizers (n = 2). The severity of social affect (SA) and RRBs was assessed using the ADOS-2 (45).

A total of 40 children with TD were recruited from the general population through advertisements and word of mouth. None of the TD participants had a history of psychiatric or neurological disorders. Screening was conducted using the Japanese version of the Autism Spectrum Quotient (AQ-J) (46, 47). TD children aged <15 years with a parent-reported AQ-J score of ≤24 (47) and those aged ≥16 years with a self-reported AQ-J score of ≤25 were included in the analysis (48). We recruited all participants between March 2021 and January 2023.

Cognitive function was assessed in all participants using the Das–Naglieri Cognitive Assessment System (DN-CAS) (49, 50). The DN-CAS is a theory-driven instrument based on the Planning, Attention, Simultaneous, and Successive cognitive processing model (51). Participants in the ASD and TD groups who obtained a total DN-CAS score of <70 and exhibited structural brain abnormalities on T1-weighted magnetic resonance imaging (MRI) were excluded.

### 2.2 Acquisition of magnetic resonance imaging and magnetic resonance spectroscopy data

**Figure 1:**
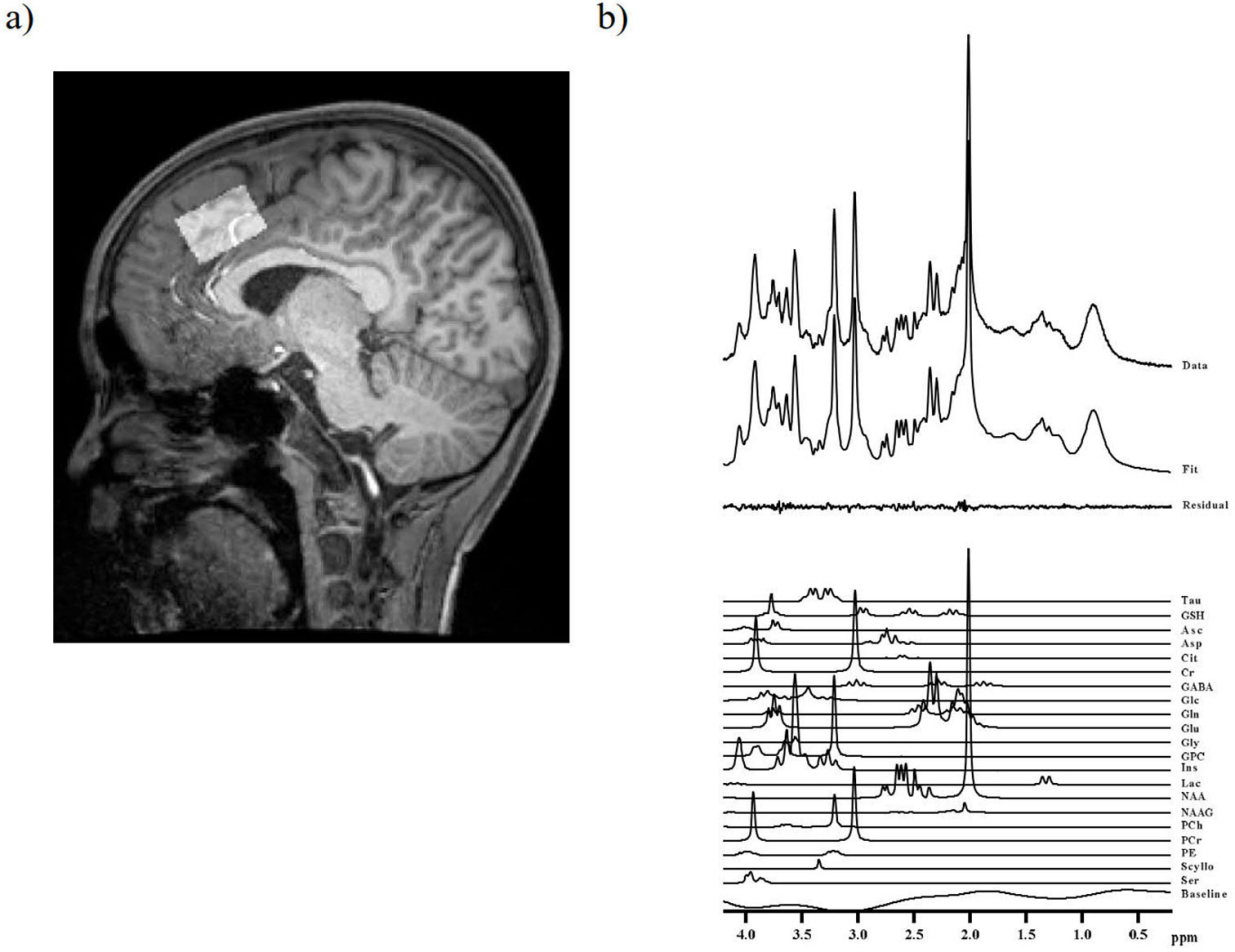
Representative VOIs, MRS spectrum, and scatterplots of metabolite levels in a child with ASD. **(A)** VOI (30 × 20 × 20 mm^3^) in the ACC of a child with ASD. ACC, anterior cingulate cortex; VOI, volume of interest; MRS, magnetic resonance spectroscopy **(B)** Representative MRS spectrum.

All MRI and MRS examinations were performed using a 3T scanner (Siemens MAGNETOM Skyra, Erlangen, Germany) equipped with a 32-channel receiver head coil. Three-dimensional volumetric images were acquired using a T1-weighted gradient-echo sequence, yielding a gapless series of thin sagittal sections (repetition time (TR) = 2,500 ms; echo time (TE) = 2.18 ms; inversion time (TI) = 1,000 ms; field of view (FOV) = 256 mm; flip angle = 8°; acquisition matrix = 320 × 300; and slice thickness = 0.8 mm). Anatomical images were used to localize the voxels of interest (VOIs) for MRS, which were positioned in the ACC (**Figure 1A**). As described in previous studies (52, 53), the MRS of the ACC was performed using a short TE spin-echo full-intensity acquired localized single-voxel spectroscopy sequence (54) with the following parameters: TE = 8.5 ms, TR = 3,000 ms, 128 averages, and VOI dimensions = 30 × 20 × 20 mm^3^. This sequence was specifically developed to acquire full-intensity signals from a defined VOI using an ultrashort TE and has been employed in previous studies to quantify GSH (53, 55). One child with ASD and three typically developing (TD) children who exhibited severe head motion were excluded from the analysis, resulting in a final sample of 44 children with ASD and 40 TD children.

### 2.3 MRS data analysis

A weighted combination of receiver channels was applied, followed by the removal of motion-corrupted averages, spectrum registration for frequency and phase drift correction, and pre-subtraction sub-spectral alignment. These preprocessing steps were conducted in MATLAB 2021a (The Mathworks, Natick, MA, USA) using the FID-A toolkit prior to signal averaging and data analysis (56). MRS data were analyzed using LCModel software version 6.3-1R (Stephen Provencher, Oakville, ON, Canada) (57), which applies linear combination modeling of the acquired spectra using simulated basis function. The neurochemical basis set included nine macromolecular functions, consistent with those employed in a previous study (52). An example of the spectrum is shown in **Figure 1B**. Metabolite concentrations were quantified using tissue water as an internal reference. To account for partial volume effects, the fractions of gray matter (GM), white matter (WM), and cerebrospinal fluid (CSF) within the VOIs were calculated by segmenting the T1-weighted images using Gannet 3.0 software (58). The assumed water concentrations for WM, GM, and CSF were 35,880 mM, 43,300 mM, and 55,556 mM, respectively (59). These values were subsequently corrected based on the partial volume fractions of GM, WM, and CSF using a previously established equation (60) and adjusted according to the T2 relaxation times specific to Tau and GSH (60, 61), as reported in previous studies. The final metabolite concentrations (μmol/g) were normalized to the combined GM and WM fractions to account for the CSF content within the VOI. We summarized the MRS methods using the MRSinMRS CHECKLIST (**Supplementary Table 1**) (62).

### 2.4 Statistical analysis

Data are expressed as mean ± standard deviation. Independent sample t-tests and χ^2^ tests were used to assess group differences in demographic characteristics and voxel-wise MRS data. Spearman’s partial rank-order correlation analyses were conducted to assess the correlations between MRS-derived metabolite levels and ADOS-2 subscale scores. All statistical analyses were performed using IBM SPSS Statistics for Windows, version 25 (IBM Corp., Armonk, NY, USA). A two-tailed p-value of <0.05 was considered significant for both group comparisons and correlation analysis.

## 3 Results

### 3.1 Participant characteristics

The demographic characteristics of the participants are summarized in **Table 1**. A total of 44 children with ASD and 40 TD children were enrolled, with ages ranging from 8 to 16 years in both groups. No significant differences were observed between the ASD and TD groups in terms of sex, age, or DN-CAS full-scale scores.

**Table 1:** Demographic and clinical characteristics of children with ASD and TD children.

Characteristic	ASD (n = 44)	TD (n = 40)	*t or χ^2^*	p
	Mean (SD), frequency (%)	Mean (SD), frequency (%)		
Sex			χ^2^ = 0.12	0.45
Female	17 (38.6)	14 (35.0)		
Male	27 (61.4)	26 (65.0)		
Age, years	12.5 (2.7)	11.9 (2.7)	*t* = 1.14	0.26
DN-CAS full scale	98.1 (13.9)	98.1 (15.2)	*t* = −0.017	0.99
ADOS-2 total scores	12.5 (2.9)	NA	NA	NA

ASD, autism spectrum disorder; ADOS-2, Autism Diagnostic Observation Schedule-Second Edition; DN-CAS, Das-Naglieri Cognitive Assessment System; SD, standard deviation; TD, typically developed.

### 3.2 Comparisons of metabolite levels between the ASD and TD groups

The spectral signal-to-noise ratio and linewidth in the ACC, as reported by LCModel, were 115.6 ± 15.5 in the ASD group and 116.4 ± 16.0 in the TD group for the signal-to-noise ratio, and 0.020 ± 0.003 ppm in the ASD group and 0.021 ± 0.004 ppm in the TD group for the linewidth (mean ± standard deviation), respectively. These results indicate excellent MRS data quality, with all measurements substantially exceeding the previously established criteria for the signal-to-noise ratio and linewidth (63). In this study, Tau and GSH levels were evaluated as markers of oxidative stress. The reliability of neurochemical quantification was evaluated using the Cramer–Rao lower bound (CRLB) provided by the LCModel for each metabolite. A CRLB cutoff of 30%, averaged across all scans, was used to determine the reliability (64). The average CRLB values for Tau and GSH were 10.6% ± 2.3% and 6.1% ± 0.9%, respectively, both of which satisfied the reliability threshold.

**Figure 2:**
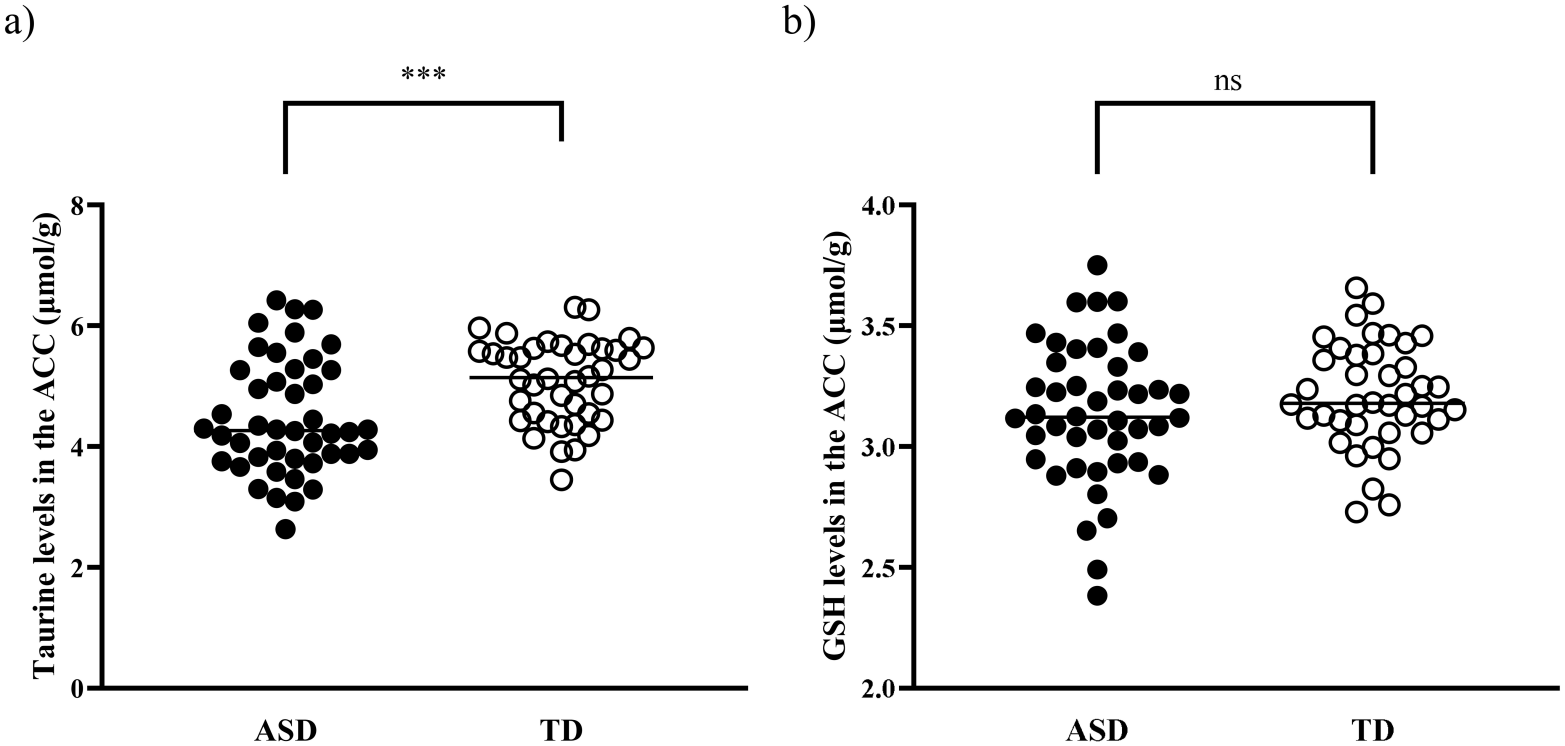
Tau and GSH levels in the ACC of children with ASD and TD children. **(A)** Tau levels in the ACC were significantly decreased in children with ASD compared with TD children (p = 0.001). **(B)** No significant difference was observed in GSH levels between the two groups (p = 0.18). ACC, anterior cingulate cortex; ASD, autism spectrum disorder; GSH, glutathione; Tau, taurine; TD, typically developed. ***p <0.005. ns, not significant.

Tau levels in the ACC were significantly lower in children with ASD than in TD controls (4.48 [0.95] for the ASD group and 5.08 [0.69] for the TD group, t = −3.36, p = 0.001) (**Figure 2**). This difference remained significant in the analysis of covariance after controlling for psychotropic medication use (F = 6.94, p = 0.010), and comorbid neuropsychiatric conditions (F = 8.66, p = 0.004). The statistical significance persisted even after excluding 18 children with ASD exposed to psychotropic medications (t = −2.39, p = 0.022), and after excluding nine children with ASD presenting with comorbid neuropsychiatric conditions (t = −2.86, p = 0.006). However, no significant difference was found in GSH levels between the two groups (3.14 [0.29] for the ASD group and 3.21 [0.22] for the TD group; t = −1.34, p = 0.18).

### 3.3 Correlations of metabolite levels with ADOS-2 scores in children with ASD

**Figure 3:**
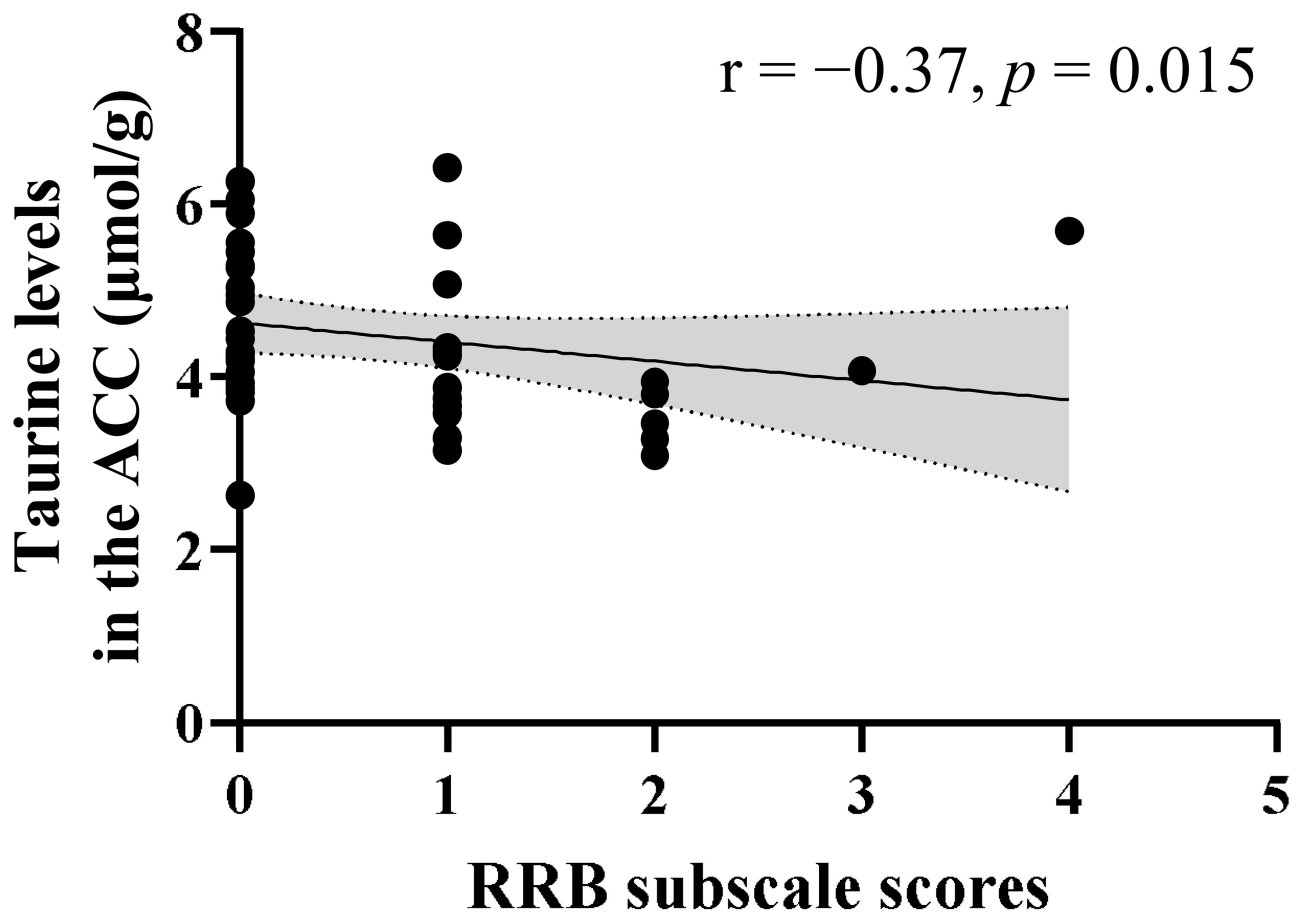
Correlation between ADOS-2 RRB scores and Tau levels in the ACC of children with ASD. A significant negative correlation was observed between ADOS-2 RRB subscale scores and Tau levels (r = −0.37, p = 0.015) in children with ASD. The shaded region indicates the 95% confidence interval. ACC, anterior cingulate cortex; ADOS-2, Autism Diagnostic Observation Schedule-Second Edition; ASD, autism spectrum disorder; RRB, restricted and repetitive behaviors; Tau, taurine.

A significant negative correlation was observed between the Tau levels in the ACC and RRB subscale scores (r = −0.37, p = 0.015) in children with ASD (**Figure 3**). Meanwhile, no significant correlation was found between the Tau levels and SA subscale scores (r = −0.16, p = 0.31). The negative correlation between Tau levels and RRB subscale scores remained significant even after controlling for psychotropic medication use as a covariate (r = −0.37, p = 0.014) and after controlling for comorbid neuropsychiatric conditions as a covariate (r = −0.38, p = 0.013). In contrast, no correlation was found between GSH levels in the ACC and either RRB (r = −0.18, p = 0.25) or SA subscale scores (r = 0.18, p = 0.24) in the ASD group.

## 4 Discussion

In this study, MRS was employed to assess the levels of Tau and GSH in the ACC of TD children and those with ASD. Significantly lower Tau levels were observed in the ACC of children with ASD than in TD controls, whereas no significant difference was found in GSH levels between the groups. Additionally, Tau levels in the ACC were significantly negatively correlated with RRB subscale scores, whereas GSH levels were not significantly correlated with autistic traits. The reliability of these findings is supported by the relatively large sample size and high spectral quality of the MRS data.

The findings of the present study are consistent with previous MRS studies (23–25) in reporting comparable GSH levels in children with ASD relative to TD controls; however, to our knowledge, this is the first study to demonstrate lower Tau levels in the ACC. Both GSH and Tau are synthesized from cysteine; however, it has been reported that the activity of cysteine sulfinate decarboxylase—an enzyme essential for Tau synthesis—is low (36). We speculate that this reduced enzymatic activity may result in comparable GSH levels and reduced Tau levels under oxidative stress in children with ASD. Furthermore, several animal studies have indicated that Tau can attenuate oxidative stress-induced reductions in GSH levels (35, 65–68). Collectively, these findings suggest that Tau may mitigate GSH depletion by exerting antioxidative effects, including scavenging free radicals (29, 30), inhibiting lipid peroxidation (31–33), and enhancing superoxide dismutase activity (34, 35).

According to the proposed hypothesis, children with ASD exhibit insufficient Tau levels in the ACC. Accumulating evidence indicates that Tau plays an important role in neurodevelopment (reviewed in (69)). Tau has been reported to function as a neurodevelopmental modulator through the GABA_A_ receptor, and alterations in GABA_A_ receptor activity during development have been shown to impair social interactions in offspring, which represent a core symptom of ASD (70). The present results are also supported by findings from both human and animal studies, which reported decreased Tau levels in ASD. For example, mice receiving gut microbiota transplants from human donors with ASD develop ASD-like behaviors and exhibit reduced Tau levels in the colon (71). Similarly, blood Tau levels have been reported to be lower in children with ASD than in TD children (40–42). The elevated urinary Tau levels observed in children with ASD (72, 73) suggest excessive urinary excretion. Moreover, restricted dietary patterns commonly associated with ASD traits (39) may result in reduced Tau intake, as this amino acid is predominantly obtained from dietary sources such as beef, chicken, fish, and shellfish (37, 38). Collectively, the combination of increased excretion and decreased dietary intake may impede Tau replenishment, which is crucial because Tau is consumed during oxidative stress.

Correlations between Tau levels in the ACC and RRB subscale were also observed in children with ASD. Tau performs numerous physiological functions in mammals (27), including antioxidant activities. In addition to its antioxidant properties, Tau has been implicated in the maintenance of neuronal functions, such as neurodevelopmental modulation (69), energy metabolism (74), inhibitory neurotransmission (75, 76), and calcium homeostasis modulation (77, 78). Tau depletion due to oxidative stress can disrupt these neuronal function. Given that the ACC has been associated with RRB in ASD (12), our findings suggest that impaired neuronal function in the ACC may be correlated with RRB. Furthermore, these findings suggest that Tau may serve as a potential pharmacological target for the treatment of RRB, which has recently been recognized as a core feature of ASD through analyses employing extended language model architectures (79). In line with this, there are studies reporting that taurine supplementation improves cognitive function and emotional behaviors in mice (80, 81), and more recently, a randomized, double-blind, placebo-controlled trial was conducted in children with ASD (82).

This study had some limitations. Despite being relatively large, the sample size of 44 children with ASD and 40 TD children may limit the generalizability of these findings. First, the participants in this study were exclusively Japanese, which might limit the generalizability of the findings to other populations. The inclusion of children with ASD who presented with comorbid conditions (83, 84) and those receiving psychotropic medications (85–87) introduced potential confounding effects on oxidative stress; nevertheless, after adjusting for these factors, taurine levels remained low in children with ASD. The exclusion of participants due to motion artifacts may have introduced a potential bias. The cross-sectional design of this study precludes the establishment of causal relationships; therefore, it remains unclear whether taurine depletion is a cause or consequence of RRBs. Moreover, age-related differences remained unaccounted for. Unmeasured factors, including dietary habits and gastrointestinal conditions, may also influence Tau and GSH levels. Restricting the analysis to the ACC may have limited the detection of broader neurochemical alterations associated with ASD. To advance the understanding of ASD, future research should be conducted with larger and more diverse samples, including multi−ethnic participants, employ longitudinal designs, and incorporate dietary assessments and a broader range of neurochemical markers.

Distinct patterns were identified in the two antioxidants examined in the ACC of children with ASD, with GSH levels remaining comparable and Tau levels significantly reduced. These findings may reflect differences in the biosynthetic pathways of GSH and Tau, and their respective roles in mitigating oxidative stress in ASD pathology. Tau depletion in the ACC may compromise its physiological functions, including its role as a neurodevelopmental modulator, potentially contributing to the manifestation of RRB in ASD. Collectively, these findings suggest that Tau may be a promising target for developing symptom-relieving therapies for ASD.

## Data Availability

The raw data supporting the conclusions of this article will be made available by the authors, without undue reservation.

## References

[1] American Psychiatric Association . Diagnostic and statistical manual of mental disorders, 5th ed. Washington, D.C., United States: American Psychiatric Association Publishing. (2013).

[2] Maenner MJ Shaw KA Baio J Washington A Patrick M DiRienzo M . Prevalence of autism spectrum disorder among children aged 8 years - autism and developmental disabilities monitoring network, 11 sites, United States, 2016. MMWR Surveill Summ. (2020) 69:1–12. doi: 10.15585/mmwr.ss6904a1, PMID: 32214087 PMC7119644

[3] Bishop-Fitzpatrick L Mazefsky CA Eack SM . The combined impact of social support and perceived stress on quality of life in adults with autism spectrum disorder and without intellectual disability. Autism. (2018) 22:703–11. doi: 10.1177/1362361317703090, PMID: 28666391 PMC5711618

[4] Lin LY . Quality of life of Taiwanese adults with autism spectrum disorder. PloS One. (2014) 9:e109567. doi: 10.1371/journal.pone.0109567, PMID: 25299379 PMC4192352

[5] Li X Zhang K He X Zhou J Jin C Shen L . Structural, functional, and molecular imaging of autism spectrum disorder. Neurosci Bull. (2021) 37:1051–71. doi: 10.1007/s12264-021-00673-0, PMID: 33779890 PMC8275699

[6] Anagnostou E Taylor MJ . Review of neuroimaging in autism spectrum disorders: what have we learned and where we go from here. Mol Autism. (2011) 2:4. doi: 10.1186/2040-2392-2-4, PMID: 21501488 PMC3102613

[7] Dichter GS Felder JN Bodfish JW . Autism is characterized by dorsal anterior cingulate hyperactivation during social target detection. Soc Cognit Affect Neurosci. (2009) 4:215–26. doi: 10.1093/scan/nsp017, PMID: 19574440 PMC2728636

[8] Balsters JH Mantini D Apps MAJ Eickhoff SB Wenderoth N . Connectivity-based parcellation increases network detection sensitivity in resting state fMRI: an investigation into the cingulate cortex in autism. NeuroImage Clin. (2016) 11:494–507. doi: 10.1016/j.nicl.2016.03.016, PMID: 27114898 PMC4832089

[9] Hassan TH Abdelrahman HM Abdel Fattah NR El-Masry NM Hashim HM El-Gerby KM . Blood and brain glutamate levels in children with autistic disorder. Res Autism Spectr Disord. (2013) 7:541–8. doi: 10.1016/j.rasd.2012.12.005

[10] Joshi G Biederman J Wozniak J Goldin RL Crowley D Furtak S . Magnetic resonance spectroscopy study of the glutamatergic system in adolescent males with high-functioning autistic disorder: a pilot study at 4T. Eur Arch Psychiatry Clin Neurosci. (2013) 263:379–84. doi: 10.1007/s00406-012-0369-9, PMID: 22986449

[11] Oya M Matsuoka K Kubota M Fujino J Tei S Takahata K . Increased glutamate and glutamine levels and their relationship to astrocytes and dopaminergic transmissions in the brains of adults with autism. Sci Rep. (2023) 13:11655. doi: 10.1038/s41598-023-38306-3, PMID: 37468523 PMC10356952

[12] Thakkar KN Polli FE Joseph RM Tuch DS Hadjikhani N Barton JJ . Response monitoring, repetitive behaviour and anterior cingulate abnormalities in autism spectrum disorders (ASD). Brain. (2008) 131:2464–78. doi: 10.1093/brain/awn099, PMID: 18550622 PMC2525446

[13] Shafritz KM Dichter GS Baranek GT Belger A . The neural circuitry mediating shifts in behavioral response and cognitive set in autism. Biol Psychiatry. (2008) 63:974–80. doi: 10.1016/j.biopsych.2007.06.028, PMID: 17916328 PMC2599927

[14] Usui N Kobayashi H Shimada S . Neuroinflammation and oxidative stress in the pathogenesis of autism spectrum disorder. Int J Mol Sci. (2023) 24:5487. doi: 10.3390/ijms24065487, PMID: 36982559 PMC10049423

[15] Bjørklund G Tinkov AA Hosnedlová B Kizek R Ajsuvakova OP Chirumbolo S . The role of glutathione redox imbalance in autism spectrum disorder: a review. Free Radic Biol Med. (2020) 160:149–62. doi: 10.1016/j.freeradbiomed.2020.07.017, PMID: 32745763

[16] Hu T Dong Y He C Zhao M He Q . The gut microbiota and oxidative stress in autism spectrum disorders (ASD). Oxid Med Cell Longev. (2020) 2020:8396708. doi: 10.1155/2020/8396708, PMID: 33062148 PMC7547345

[17] Membrino V Di Paolo A Alia S Papiri G Vignini A . The role of oxidative stress in autism spectrum disorder: a narrative literature review. Oxygen. (2023) 3:34–44. doi: 10.3390/oxygen3010004

[18] Estes ML McAllister AK . Maternal immune activation: implications for neuropsychiatric disorders. Science. (2016) 353:772–7. doi: 10.1126/science.aag3194, PMID: 27540164 PMC5650490

[19] Birben E Sahiner UM Sackesen C Erzurum S Kalayci O . Oxidative stress and antioxidant defense. World Allergy Organ J. (2012) 5:9–19. doi: 10.1097/WOX.0b013e3182439613, PMID: 23268465 PMC3488923

[20] Uddin MN Mondal T Yao Y Manley K Lawrence DA . Oxidative stress and neuroimmune proteins in a mouse model of autism. Cell Stress Chaperones. (2023) 28:201–17. doi: 10.1007/s12192-023-01331-2, PMID: 36795226 PMC10050529

[21] Frustaci A Neri M Cesario A Adams JB Domenici E Dalla Bernardina B . Oxidative stress-related biomarkers in autism: systematic review and meta-analyses. Free Radic Biol Med. (2012) 52:2128–41. doi: 10.1016/j.freeradbiomed.2012.03.011, PMID: 22542447

[22] Thomson AR Pasanta D Arichi T Puts NA . Puts, neurometabolite differences in autism as assessed with magnetic resonance spectroscopy: a systematic review and meta-analysis. Neurosci Biobehav Rev. (2024) 162:105728. doi: 10.1016/j.neubiorev.2024.105728, PMID: 38796123 PMC11602446

[23] Durieux AMS Horder J Mendez MA Egerton A Williams SCR Wilson CE . Cortical and subcortical glutathione levels in adults with autism spectrum disorder. Autism Res. (2016) 9:429–35. doi: 10.1002/aur.1522, PMID: 26290215 PMC4761328

[24] Endres D Tebartz van Elst L Meyer SA Feige B Nickel K Bubl A . Glutathione metabolism in the prefrontal brain of adults with high-functioning autism spectrum disorder: an MRS study. Mol Autism. (2017) 8:10. doi: 10.1186/s13229-017-0122-3, PMID: 28316774 PMC5351053

[25] Sapey-Triomphe LA Temmerman J Puts NAJ Wagemans J . Prediction learning in adults with autism and its molecular correlates. Mol Autism. (2021) 12:64. doi: 10.1186/s13229-021-00470-6, PMID: 34615532 PMC8493731

[26] Song Y Hupfeld KE Davies-Jenkins CW Zöllner HJ Murali-Manohar S Mumuni AN . Brain Glutathione and GABA+ levels in autistic children. bioRxiv. (2023). doi: 10.1101/2023.09.28.559718, PMID: 38279628 PMC10963146

[27] Schaffer S Kim HW . Effects and mechanisms of taurine as a therapeutic agent. Biomol Ther (Seoul). (2018) 26:225–41. doi: 10.4062/biomolther.2017.251, PMID: 29631391 PMC5933890

[28] Surai PF Earle-Payne K Kidd MT . Taurine as a natural antioxidant: from direct antioxidant effects to protective action in various toxicological models. Antioxidants (Basel). (2021) 10:1876. doi: 10.3390/antiox10121876, PMID: 34942978 PMC8698923

[29] Aruoma OI Halliwell B Hoey BM Butler J . The antioxidant action of taurine, hypotaurine and their metabolic precursors. Biochem J. (1988) 256:251–5. doi: 10.1042/bj2560251, PMID: 2851980 PMC1135395

[30] Oliveira MW Minotto JB de Oliveira MR Zanotto-Filho A Behr GA Rocha RF . Scavenging and antioxidant potential of physiological taurine concentrations against different reactive oxygen/nitrogen species. Pharmacol Rep. (2010) 62:185–93. doi: 10.1016/s1734-1140(10)70256-5, PMID: 20360629

[31] Nandhini AT Thirunavukkarasu V Ravichandran MK Anuradha CV . Effect of taurine on biomarkers of oxidative stress in tissues of fructose-fed insulin-resistant rats. Singapore Med J. (2005) 46:82–7., PMID: 15678290

[32] Goodman CA Horvath D Stathis C Mori T Croft K Murphy RM . Taurine supplementation increases skeletal muscle force production and protects muscle function during and after high-frequency *in vitro* stimulation. J Appl Physiol. (2009) 107:144–54. doi: 10.1152/japplphysiol.00040.2009, PMID: 19423840 PMC2711783

[33] Parvez S Tabassum H Banerjee BD Raisuddin S . Taurine prevents tamoxifen-induced mitochondrial oxidative damage in mice. Basic Clin Pharmacol Toxicol. (2008) 102:382–7. doi: 10.1111/j.1742-7843.2008.00208.x, PMID: 18312495

[34] Nonaka H Tsujino T Watari Y Emoto N Yokoyama M . Taurine prevents the decrease in expression and secretion of extracellular superoxide dismutase induced by homocysteine: amelioration of homocysteine-induced endoplasmic reticulum stress by taurine. Circulation. (2001) 104:1165–70. doi: 10.1161/hc3601.093976, PMID: 11535574

[35] Jafri AJA Agarwal R Iezhitsa I Agarwal P Ismail NM . Taurine protects against NMDA-induced retinal damage by reducing retinal oxidative stress. Amino Acids. (2019) 51:641–6. doi: 10.1007/s00726-019-02696-4, PMID: 30656415

[36] Huxtable RJ . Expanding the circle 1975–1999: sulfur biochemistry and insights on the biological functions of taurine. Adv Exp Med Biol. (2000) 483:1–25. doi: 10.1007/0-306-46838-7_1, PMID: 11787586

[37] Kadam SU Prabhasankar P . Marine foods as functional ingredients in bakery and pasta products. Food Res Int. (2010) 43:1975–80. doi: 10.1016/j.foodres.2010.06.007

[38] Laidlaw SA Grosvenor M Kopple JD . The taurine content of common foodstuffs. JPEN J Parenter Enteral Nutr. (1990) 14:183–8. doi: 10.1177/0148607190014002183, PMID: 2352336

[39] Byrska A Błażejczyk I Faruga A Potaczek M Wilczyński KM Janas-Kozik M . Patterns of food selectivity among children with autism spectrum disorder. J Clin Med. (2023) 12:5469. doi: 10.3390/jcm12175469, PMID: 37685537 PMC10488249

[40] Tu WJ Chen H He J . Application of LC-MS/MS analysis of plasma amino acids profiles in children with autism. J Clin Biochem Nutr. (2012) 51:248–9. doi: 10.3164/jcbn.12-45, PMID: 23170055 PMC3491252

[41] Adams JB Audhya T McDonough-Means S Rubin RA Quig D Geis E . Nutritional and metabolic status of children with autism vs. neurotypical children, and the association with autism severity. Nutr Metab. (2011) 8:34. doi: 10.1186/1743-7075-8-34, PMID: 21651783 PMC3135510

[42] Geier DA Kern JK Garver CR Adams JB Audhya T Geier MR . A prospective study of transsulfuration biomarkers in autistic disorders. Neurochem Res. (2009) 34:386–93. doi: 10.1007/s11064-008-9782-x, PMID: 18612812

[43] Shimmura C Suda S Tsuchiya KJ Hashimoto K Ohno K Matsuzaki H . Alteration of plasma glutamate and glutamine levels in children with high-functioning autism. PloS One. (2011) 6:e25340. doi: 10.1371/journal.pone.0025340, PMID: 21998651 PMC3187770

[44] Bruce MR Couch ACM Grant S McLellan J Ku K Chang C . Altered behavior, brain structure, and neurometabolites in a rat model of autism-specific maternal autoantibody exposure. Mol Psychiatry. (2023) 28:2136–47. doi: 10.1038/s41380-023-02020-3, PMID: 36973347 PMC10575787

[45] Lord C Rutter M DiLavore PC Risi S Gotham K Bishop S . Autism diagnostic observation schedule. 2nd ed. Torrance, CA: Western Services (2012).

[46] Baron-Cohen S Wheelwright S Skinner R Martin J Clubley E . The autism-spectrum quotient (AQ): evidence from Asperger syndrome/high-functioning autism, males and females, scientists and mathematicians. J Autism Dev Disord. (2001) 31:5–17. doi: 10.1023/a:1005653411471, PMID: 11439754

[47] Kurita H Koyama T Osada H . Osada, Autism-Spectrum Quotient-Japanese version and its short forms for screening normally intelligent persons with pervasive developmental disorders. Psychiatry Clin Neurosci. (2005) 59:490–6. doi: 10.1111/j.1440-1819.2005.01403.x, PMID: 16048456

[48] Wakabayashi A Uchiyama T Tojo Y Yoshida Y Kuroda M Baron-Cohen S . Autism-spectrum quotient (AQ) Japanese children’s version comparison between high-functioning children with autism spectrum disorders and normal controls. Shinrigaku Kenkyu. (2007) 77:534–40. doi: 10.4992/jjpsy.77.534, PMID: 17447462

[49] Maekawa H Okayama S . Japanese version of the das-naglieri cognitive assessment system. Tokyo: Nihon Bunka Kagakusha. (2007).

[50] Naglieri JA . The essentials of CAS assessment. New York: Wiley. (1999).

[51] Pérez-Alvarez F Timoneda-Gallart C . PASS neurocognitive dysfunction in attention deficit. Rev Neurol. (2001) 32:30–7. 11293095

[52] Hirata K Matsuoka K Tagai K Endo H Tatebe H Ono M . brain energy metabolism related to astrocytes in Alzheimer’s disease. Ann Neurol. (2023) 95:104–15. doi: 10.1002/ana.26797, PMID: 37703428

[53] Matsuoka K Takado Y Tagai K Kubota M Sano Y Takahata K . Two pathways differentially linking tau depositions, oxidative stress, and neuronal loss to apathetic phenotypes in progressive supranuclear palsy. J Neurol Sci. (2023) 444:120514. doi: 10.1016/j.jns.2022.120514, PMID: 36473346

[54] Mekle R Mlynárik V Gambarota G Hergt M Krueger G Gruetter R . MR spectroscopy of the human brain with enhanced signal intensity at ultrashort echo times on a clinical platform at 3T and 7T. Magn Reson Med. (2009) 61:1279–85. doi: 10.1002/mrm.21961, PMID: 19319893

[55] Xin L Mekle R Fournier M Baumann PS Ferrari C Alameda L . Genetic polymorphism associated prefrontal glutathione and its coupling with brain glutamate and peripheral redox status in early psychosis. Schizophr Bull. (2016) 42:1185–96. doi: 10.1093/schbul/sbw038, PMID: 27069063 PMC4988744

[56] Simpson R Devenyi GA Jezzard P Hennessy TJ Near J . Advanced processing and simulation of MRS data using the FID appliance (FID-A)-An open source, MATLAB-based toolkit. Magn Reson Med. (2017) 77:23–33. doi: 10.1002/mrm.26091, PMID: 26715192

[57] Provencher SW . Estimation of metabolite concentrations from localized *in vivo* proton NMR spectra. Magn Reson Med. (1993) 30:672–9. doi: 10.1002/mrm.1910300604, PMID: 8139448

[58] Harris AD Puts NA Edden RA . Tissue correction for GABA-edited MRS: considerations of voxel composition, tissue segmentation, and tissue relaxations. J Magn Reson Imaging. (2015) 42:1431–40. doi: 10.1002/jmri.24903, PMID: 26172043 PMC4615266

[59] Ernst T Kreis R Ross BD . Absolute quantitation of water and metabolites in the human brain. I. Compartments and water. J Magnetic Resonance Ser B. (1993) 102:1–8. doi: 10.1006/jmrb.1993.1055

[60] Dhamala E Abdelkefi I Nguyen M Hennessy TJ Nadeau H Near J . Validation of *in vivo* MRS measures of metabolite concentrations in the human brain. NMR BioMed. (2019) 32:e4058. doi: 10.1002/nbm.4058, PMID: 30663818

[61] Wyss PO Bianchini C Scheidegger M Giapitzakis IA Hock A Fuchs A . *In vivo* estimation of transverse relaxation time constant (T2) of 17 human brain metabolites at 3T. Magn Reson Med. (2018) 80:452–61. doi: 10.1002/mrm.27067, PMID: 29344979

[62] Lin A Andronesi O Bogner W Choi IY Coello E Cudalbu C . Minimum Reporting Standards for *in vivo* Magnetic Resonance Spectroscopy (MRSinMRS): Experts’ consensus recommendations. NMR BioMed. (2021) 34:e4484. doi: 10.1002/nbm.4484, PMID: 33559967 PMC8647919

[63] Wilson M Andronesi O Barker PB Bartha R Bizzi A Bolan PJ . Methodological consensus on clinical proton MRS of the brain: review and recommendations. Magn Reson Med. (2019) 82:527–50. doi: 10.1002/mrm.27742, PMID: 30919510 PMC7179569

[64] Chowdhury FA O’Gorman RL Nashef L Elwes RD Edden RA Murdoch JB . Investigation of glutamine and GABA levels in patients with idiopathic generalized epilepsy using MEGAPRESS. J Magn Reson Imaging. (2015) 41:694–9. doi: 10.1002/jmri.24611, PMID: 24585443 PMC4407645

[65] Oudit GY Trivieri MG Khaper N Husain T Wilson GJ Liu P . Taurine supplementation reduces oxidative stress and improves cardiovascular function in an iron-overload murine model. Circulation. (2004) 109:1877–85. doi: 10.1161/01.cir.0000124229.40424.80, PMID: 15037530

[66] Pushpakiran G Mahalakshmi K Anuradha CV . Protective effects of taurine on glutathione and glutathione-dependent enzymes in ethanol-fed rats. Pharmazie. (2004) 59:869–72., PMID: 15587589

[67] Hagar HH . The protective effect of taurine against cyclosporine A-induced oxidative stress and hepatotoxicity in rats. Toxicol Lett. (2004) 151:335–43. doi: 10.1016/j.toxlet.2004.03.002, PMID: 15183458

[68] Sener G Ozer Sehirli A Ipçi Y Cetinel S Cikler E Gedik N . Taurine treatment protects against chronic nicotine-induced oxidative changes. Fundam Clin Pharmacol. (2005) 19:155–64. doi: 10.1111/j.1472-8206.2005.00322.x, PMID: 15810895

[69] Furukawa T Fukuda A . Maternal taurine as a modulator of Cl(-) homeostasis as well as of glycine/GABA(A) receptors for neocortical development. Front Cell Neurosci. (2023) 17:1221441. doi: 10.3389/fncel.2023.1221441, PMID: 37601283 PMC10435090

[70] Tochitani S Furukawa T Bando R Kondo S Ito T Matsushima Y . GABAA receptors and maternally derived taurine regulate the temporal specification of progenitors of excitatory glutamatergic neurons in the mouse developing cortex. Cereb Cortex. (2021) 31:4554–75. doi: 10.1093/cercor/bhab106, PMID: 34013343

[71] Sharon G Cruz NJ Kang DW Gandal MJ Wang B Kim YM . Human gut microbiota from autism spectrum disorder promote behavioral symptoms in mice. Cell. (2019) 177:1600–18.e1617. doi: 10.1016/j.cell.2019.05.004, PMID: 31150625 PMC6993574

[72] Nadal-Desbarats L Aïdoud N Emond P Blasco H Filipiak I Sarda P . Combined 1H-NMR and 1H-13C HSQC-NMR to improve urinary screening in autism spectrum disorders. Analyst. (2014) 139:3460–8. doi: 10.1039/c4an00552j, PMID: 24841505

[73] Yap IK Angley M Veselkov KA Holmes E Lindon JC Nicholson JK . Urinary metabolic phenotyping differentiates children with autism from their unaffected siblings and age-matched controls. J Proteome Res. (2010) 9:2996–3004. doi: 10.1021/pr901188e, PMID: 20337404

[74] Schaffer SW Shimada-Takaura K Jong CJ Ito T Takahashi K . Impaired energy metabolism of the taurine−deficient heart. Amino Acids. (2016) 48:549–58. doi: 10.1007/s00726-015-2110-2, PMID: 26475290

[75] El Idrissi A Messing J Scalia J Trenkner E . Prevention of epileptic seizures by taurine. Adv Exp Med Biol. (2003) 526:515–25. doi: 10.1007/978-1-4615-0077-3_62, PMID: 12908638

[76] Quinn MR Harris CL . Taurine allosterically inhibits binding of [35S]-t-butylbicyclophosphorothionate (TBPS) to rat brain synaptic membranes. Neuropharmacology. (1995) 34:1607–13. doi: 10.1016/0028-3908(95)00118-2, PMID: 8788958

[77] Ramila KC Jong CJ Pastukh V Ito T Azuma J Schaffer SW . Role of protein phosphorylation in excitation-contraction coupling in taurine deficient hearts. Am J Physiol Heart Circ Physiol. (2015) 308:H232–9. doi: 10.1152/ajpheart.00497.2014, PMID: 25437920

[78] Wu H Jin Y Wei J Jin H Sha D Wu JY . Mode of action of taurine as a neuroprotector. Brain Res. (2005) 1038:123–31. doi: 10.1016/j.brainres.2005.01.058, PMID: 15757628

[79] Stanley J Rabot E Reddy S Belilovsky E Mottron L Bzdok D . Large language models deconstruct the clinical intuition behind diagnosing autism. Cell. (2025) 188:2235–48.e2210. doi: 10.1016/j.cell.2025.02.025, PMID: 40147442

[80] Ommati MM Rezaei H Socorro RM Tian W Zhao J Rouhani A . Pre/postnatal taurine supplementation improves neurodevelopment and brain function in mice offspring: A persistent developmental study from puberty to maturity. Life Sci. (2024) 336:122284. doi: 10.1016/j.lfs.2023.122284, PMID: 38008208

[81] Rezaei H Wang HW Tian W Zhao J Najibi A Retana-Márquez S . Long-term taurine supplementation regulates brain mitochondrial dynamics in mice. Basic Clin Pharmacol Toxicol. (2025) 136:e14101. doi: 10.1111/bcpt.14101, PMID: 39558449

[82] Chen Y He W Deng Q Peng Z Tai Z Ma Y . Taurine supplementation in children with autism spectrum disorders: a study protocol for an exploratory randomized, double-blind, placebo-controlled trial. BMC Pediatr. (2025) 25:871. doi: 10.1186/s12887-025-06216-0, PMID: 41146076 PMC12560396

[83] Brennan BP Jensen JE Perriello C Pope HG Jr. Jenike MA Hudson JI . LOWER POSTERIOR CINGULATE CORTEX GLUTATHIONE LEVELS IN OBSESSIVE-COMPULSIVE DISORDER. Biol Psychiatry Cognit Neurosci Neuroimaging. (2016) 1:116–24. doi: 10.1016/j.bpsc.2015.12.003, PMID: 26949749 PMC4774044

[84] Nasim S Naeini AA Najafi M Ghazvini M Hassanzadeh A . Relationship between antioxidant status and attention deficit hyperactivity disorder among children. Int J Prev Med. (2019) 10:41. doi: 10.4103/ijpvm.IJPVM_80_18, PMID: 31057726 PMC6484508

[85] Foschiera LN Schmitz F Wyse ATS . Evidence of methylphenidate effect on mitochondria, redox homeostasis, and inflammatory aspects: insights from animal studies. Prog Neuropsychopharmacol Biol Psychiatry. (2022) 116:110518. doi: 10.1016/j.pnpbp.2022.110518, PMID: 35092763

[86] Kośmider K Kamieniak M Czuczwar SJ Miziak B . Second generation of antiepileptic drugs and oxidative stress. Int J Mol Sci. (2023) 24:3873. doi: 10.3390/ijms24043873, PMID: 36835284 PMC9964930

[87] Ribaudo G Bortoli M Pavan C Zagotto G Orian L . Antioxidant potential of psychotropic drugs: from clinical evidence to *in vitro* and *in vivo* assessment and toward a new challenge for in silico molecular design. Antioxidants (Basel). (2020) 9:714. doi: 10.3390/antiox9080714, PMID: 32781750 PMC7465375

