# Supplementary material for: Association between decreased taurine levels in the anterior cingulate cortex and restricted and repetitive behaviors in autism spectrum disorder: a cross-sectional study
Source: Front Psychiatry. 2025 Dec 11;16:1700059. doi: 10.3389/fpsyt.2025.1700059 (PMC12739181; doi:10.3389/fpsyt.2025.1700059)
Supplement: Supplementary file 1 [file Table1.docx]

Supplementary materials

Supplementary Table 1. The MRSinMRS CHECKLIST

| **Supplementary Table 1. The MRSinMRS CHECKLIST** | |
| --- | --- |
| **1. Hardware** |  |
| a. Field strength [T] | 3 T |
| b. Manufacturer | Siemens |
| c. Model (software version if available) | Skyra (VE11) |
| d. RF coils: nuclei (transmit/receive), number of channels, type, body part | 32-channel head coil |
| e. Additional hardware | N/A |
| **2. Acquisition** |  |
| a. Pulse sequence | a short TE spin-echo full-intensity acquired localized single-voxel spectroscopy (SPECIAL) sequence |
| b. Volume of interest (VOI) locations | Anterior cingulate cortex |
| c. Nominal VOI size [cm^3^, mm^3^] | 30 × 20 × 20 mm^3^ |
| d. Repetition time (*T_R_*), echo time (*T_E_*) [ms, s] | *T_R_* = 3000 ms, *T_E_* = 8.5 ms |
| e. Total number of excitations or acquisitions per spectrum In time series for kinetic studies i. Number of averaged spectra (NA) per time point ii. Averaging method (eg block-wise or moving average) iii. Total number of spectra (acquired/in time series) | 128 averages |
| f. Additional sequence parameters (spectral width in Hz, number of spectral points, frequency offsets). If STEAM:, mixing time (*T_M_*). If MRSI: 2D or 3D, FOV in all directions, matrix size, acceleration factors, sampling method | spectral width 2000 Hz |
| g. Water suppression method | VAPOR |
| h. Shimming method, reference peak, and thresholds for “acceptance of shim” chosen | Automated B0 field mapping followed by manual shimming of water to < 14 Hz |
| i. Triggering or motion correction method (respiratory, peripheral, cardiac triggering, incl. device used and delays) | N/A |
| **3. Data analysis methods and outputs** |  |
| a. Analysis software | LCmodel vers 6.3 |
| b. Processing steps deviating from quoted reference or product | Custom basis set |
| c. Output measure (eg absolute concentration, institutional units, ratio), processing steps deviating from quoted reference or product | absolute concentration |
| d. Quantification references and assumptions, fitting model assumptions | We used a neurochemical basis set that incorporated 9 macromolecular basis functions, each sourced from the individual peaks in a macromolecular spectrum obtained by the summation of experimentally acquired metabolite-nulled spectra from 6 healthy adult volunteers. |
| **4. Data quality** |  |
| a. Reported variables (SNR, linewidth (with reference peaks)) | SNR: 116.0 ± 15.7 FWHM: 0.020 ± 0.004 ppm as reported by LCmodel |
| b. Data exclusion criteria | SNR < 5 or FWHM > 0.143 ppm |
| c. Quality measures of postprocessing model fitting (eg CRLB, goodness of fit, SD of residual) | CRLB < 30% |
| d. Sample spectrum | Figure 1 |
